# Hydrophobic Leather Coating for Footwear Applications by a Low-Pressure Plasma Polymerisation Process

**DOI:** 10.3390/polym13203549

**Published:** 2021-10-14

**Authors:** Carlos Ruzafa Silvestre, María Pilar Carbonell Blasco, Saray Ricote López, Henoc Pérez Aguilar, María Ángeles Pérez Limiñana, Elena Bañón Gil, Elena Orgilés Calpena, Francisca Arán Ais

**Affiliations:** Footwear Technology Centre, Campo Alto Campground, 03600 Alicante, Spain; pcarbonell@inescop.es (M.P.C.B.); sricote@inescop.es (S.R.L.); hperez@inescop.es (H.P.A.); maperez@inescop.es (M.Á.P.L.); elenab@inescop.es (E.B.G.); eorgiles@inescop.es (E.O.C.); aran@inescop.es (F.A.A.)

**Keywords:** low-pressure plasma, plasma process, plasma polymerisation, surface modification, plasma deposition

## Abstract

The aim of this work is to develop hydrophobic coatings on leather materials by plasma polymerisation with a low-pressure plasma system using an organosilicon compound, such as hexamethyldisiloxane (HMDSO), as chemical precursor. The hydrophobic coatings obtained by this plasma process were evaluated with different experimental techniques such as Fourier transform infrared spectroscopy (FTIR), X-ray photoelectron spectroscopy (XPS), scanning electron microscopy (SEM) and standardised tests including colour measurements of the samples, surface coating thickness and water contact angle (WCA) measurements. The results obtained indicated that the monomer had polymerised correctly and completely on the leather surface creating an ultra-thin layer based on polysiloxane. The surface modification produced a water repellent effect on the leather that does not alter the visual appearance and haptic properties. Therefore, the application of the plasma deposition process showed promising results that makes it a more sustainable alternative to conventional functional coatings, thus helping to reduce the use of hazardous chemicals in the finishing process of footwear manufacturing.

## 1. Introduction

Leather is a natural material commonly used to produce a wide range of footwear. Depending on the type of footwear and its intended use, a different type of leather with specific features is required. For such purpose during post-tanning operations, numerous finishing additives and treatments are used to improve certain leather properties, such as water resistance, oleophobicity, perspirability, flame retardancy, antimicrobial properties and abrasion resistance or antistatic properties [1].

Specifically, leather is a hydrophilic material due to the polar groups of the collagen fibers’ amino acid residues that promote their interaction with water molecules. However, this property is not desirable in certain applications where water-resistant leather is required. This repellence can be achieved by a combination of the material’s structure and finish with specific chemicals such as fluorocarbons, wax emulsions, metallic soaps and surface-active agents [2,3,4].

More durable waterproofing treatments are mainly based on halocarbons compounds, including chlorofluorocarbons (CFCs), perfluorocarbons (PFCs), hydrofluorocarbons (HFCs) and hydrochlorofluorocarbons (HCFCs), which are considered as greenhouse gases (GHGs), since they absorb infrared radiation emitted by the Earth in a spectral range where the energy is not removed by CO_2_ or water vapour [5].

For instance, HCFCs have been used to replace CFCs in several applications because they have a shorter atmospheric lifetime, and consequently, cause less ozone depletion. HFCs and PFCs have also been identified as potential replacements for ozone depleting substances (ODS) in the long term since they do not contain bromine or chlorine and do not cause significant ozone depletion. However, all these substances are also GHGs, and contribute thus to varying degrees to climate change [5]. For this reason, there is a high concern about the continued release of these persistent organic pollutants (POPs) into the environment [6], being also bioacumulative chemicals. That is why most companies are looking for alternatives to replace not only the compounds used for waterproofing but also the chemical process used to do so [7,8,9,10,11], to reduce not only the input of materials into the process but also the production of waste, contributing thus to reduce environmental footprint of the process [12].

Some alternative processes to traditional ones are currently based on plasma technologies as they are more resource-efficient and sustainable processes. These technologies might imply a significant reduction in the environmental impacts of traditional coating processes in terms of greenhouse gas emissions, reduction of hazardous chemicals and waste, and water and energy consumption [13].

Specifically, the plasma polymerisation technology can infuse monomers into plasma and coat surfaces with ultra-thin layers, such as polymer coatings, by formation of gas-phase free radicals and their recombination at radical sites during film growth resulting in stable and durable structures [14] able to impart functional properties to a great variety of materials. Many monomers can be used for the deposition of ultra-thin films by plasma polymerisation, among which the most widely used is hexamethyldisiloxane (HMDSO) [9,10,11,15,16].

Different studies focused on plasma polymerisation of HMDSO to obtain super hydrophobic and water repellency properties to different materials. They reported that the achieved film properties depend on the plasma composition [6,7] and the polymerisation process conditions, such as plasma power and monomer flux [8,9,10,11,12,13,14,15,16,17]. Nevertheless, little work has been carried out on plasma deposition on natural leathers.

In addition, Thomas Gengenbach and Hans Griesser conducted extensive studies on the generation of hydrophobic coatings by low-pressure plasma for various polymer, glass and silicon substrates, and also in the electronics sector. The aforementioned studies have been summarised by Siow et al., which includes the works from the 1980s as well as those from electronic sectors [18]. The role of surface restructuring in the ageing behaviour of siloxane plasma polymer was discussed by Gengenbach and Griesser in their study of HMDSO and hexamethyldisilazane (HMDSN) plasma polymers [19]. In another study, they concluded that the perfluoro-1,3-dimethylcyclohexane (PFDMCH) plasma polymers were therefore well deposited and suited for applications that require long-term stability and predictable, stable interfacial properties [20].

In this sense, a previous work carried out by the authors was based on the use of Multiple Laser Surface Enhancement (MLSE) technology in the framework of the European Life Textileather project [13], to provide leathers and textiles with functional properties such as water, fire and stain resistances, as well as antimicrobial properties. MLSE technology combines atmospheric plasma and laser treatments in the presence of non-toxic gases, such as nitrogen (N_2_) and oxygen (O_2_), allowing the surface modification of materials [21]. This treatment produces nanometric scale modifications, which enables the functionalisation of the material without noticeably affecting its appearance. In addition, Kayaoglu et al. reported surface modifications of natural leather samples through plasma polymerisation of different HMDSO/toluene compositions at low pressure that showed promising results towards improving the easy clean property of natural leather in upholstery applications [14]. Gaidau et al. also reported the use of cold plasma generated by dielectric barrier discharge (DBD) at atmospheric pressure and room temperature as a promising technique for dry reactivation and physical modification of collagen and keratin-based materials to improve complex characteristics, such as water repellence and flame retardancy [22]. Finally, a concise overview on plasma treatment for application on textile and leather materials was provided recently by Tudoran et al. [23].

In the present study, a low-pressure plasma system was used for the development of hydrophobic coating by plasma polymerisation on natural leather for footwear applications. For the coating treatments, the chemical precursor 100% hexamethyldisiloxane (HMDSO) was selected because it contains silicon groups, which can be favourable for improving the hydrophobic properties of the materials. For this purpose, a plasma process involving consecutive activation and etching using oxygen as the reactive gas, and HMDSO polymerisation using argon as the inert gas, has been carried out. The properties of the thin film deposited on the leather surface were characterised by different experimental techniques, in terms of water repellence, surface modifications, thickness and colour and appearance changes. The aim of this research is to use plasma treatment as an environmentally friendly finishing method to impart hydrophobicity to the surface of natural leather, which can be used in the footwear and leather industries as well as in the textile industry [24,25,26,27,28,29,30,31,32].

## 2. Experimental

### 2.1. Materials

In this work, hexamethyldisiloxane (HMDSO, (CH_3_)_3_-Si-O-Si-(CH_3_)_3_, 98% purity) provided by Merck Life Science S.L.U. (Madrid, Spain) was used as a hydrophobic monomeric precursor, as received. For plasma polymerisation processes, a blue chrome-tanned cow leather with aniline finishing supplied by the company Pies Cuadrados Leather S.L. (Aspe, Spain) was used as representative upper material for footwear applications. Table 1 includes the main physical and mechanical properties of the used leather.

Argon (Ar) and oxygen (O_2_) were used as carrier gases, both with 99.995% purity and supplied by Carburos Metálicos S.A. (Barcelona, Spain).

### 2.2. Plasma Treatment

Plasma polymerised HMDSO films on leather samples were prepared in a nano low-pressure plasma equipment (Diener Electronic Vertriebs GmbH, Germany), of modular configuration, with a chamber volume of 24 L, in stainless steel and with a tray for sample support. The plasma reactor was operating at a fixed frequency of 13.56 MHz and 300 W of maximum power. All the samples in this work were treated at the plasma power of 150 W. A composition of 100% HMDSO was injected by a micro-dossing pump during plasma polymerisation on natural leather samples.

This low-pressure plasma system is equipped with two gas supply channels. Oxygen (O_2_) and argon (Ar) were used as working gases with flows at 500 mL/min and 685 mL/min, respectively. A micro-dosing pump introduces the monomer into the reactor at 0.5 µL/s. The thermostatised treatment chamber allows the temperature to be raised during the plasma process, which increases the effectiveness of the deposition process [23,33]. (Table 2).

The treatment comprises different and consecutive stages: activation, polymerisation and etching.

Firstly, activation with plasma was carried out to create radical sites in which HMDSO reacts, and greatly facilitated thus the adhesion of non-reactive or non-wettable surfaces. The type of mechanisms that primarily contribute to the activation effect depend on the material to be treated and the nature of the plasma gas. As a working gas, oxygen was used to provide leather with new surface functionalities that completely reverse the polarity of leather samples increasing its surface wettability to improve the HMDSO deposition. In addition to these factors, the working pressure, the power of the plasma and the activation time of the gas used are also crucial.

Secondly, HMDSO was added to produce its polymerisation by plasma. This monomer is in a liquid state under ambient conditions, and when subjected to vacuum conditions, it becomes gaseous. This causes it to be susceptible to being excited by an energy source, which gives rise to silicon radicals and atoms of silicon, hydrogen, carbon and oxygen that interact on the surface of the treated material. Consequently, an ultra-thin coating layer is deposited onto leather surface permanently. For this stage, argon was selected as conductive gas because it is an inert gas and does not react with the monomeric precursor. It has a large mechanical effect since it continuously removes individual atoms from the surface contributing to a suitable layer anchorage. The properties provided by the newly deposited layer will depend on both the nature of the monomer used and the different conditions used during the process. The parameters that greatly influence the surface finish and therefore determine the water repellence obtained are the pump flow, the conductive gas, the selected prepolymer, the coating time and the number of coats applied, among others [34].

After the polymerisation stage, a fast-etching process was conducted with oxygen gas to strengthen the polymerised HMDSO layer anchorage and achieve coatings with a considerable and effective thickness.

**Figure 1:**
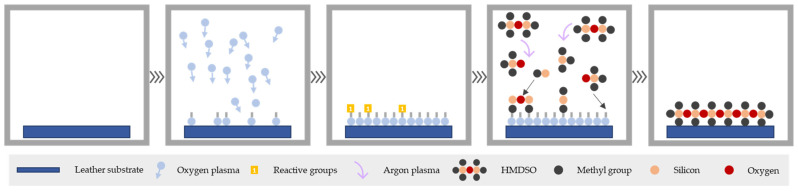
Surface activation and plasma polymerisation phases of the treatment.

Finally, three HMDSO layers were deposited onto leather samples surface with argon gas according to the operating conditions described in Table 2, which were previously optimised in the framework of the COATPLAS project [35,36]. In addition, Figure 1 summarises the process conducted in this work.

Samples with and without plasma treatment will be referenced as “plasma coated leather” and “untreated leather”, respectively.

### 2.3. Fourier Transform Infrared Spectroscopy (FTIR)

The surface chemical modifications of the coated leather were determined using a Varian 660-IR infrared spectrophotometer (VARIAN Australia PTY LTD; Mulgrave, Australia). Attenuated total reflectance (ATR) mode with 16 scans at a resolution of 4 cm^−1^ was used as the FTIR sampling technique. This ATR accessory works by measuring changes in the infrared beam when the beam comes into contact with the sample.

### 2.4. X-Ray Photoelectron Spectroscopy (XPS)

An X-ray Photoelectron Spectroscopy (XPS, K-ALPHA, Thermo Scientific) was used to analyse the chemical compositions of the surface of the siloxane polymer film obtained by plasma. Due to these films being extremely thin, XPS is the most suitable technique to determine their chemical properties. This analysis was conducted by the Technical Research Services (SSTTI) of the University of Alicante (UA). XPS measurements were collected with K-ALPHA (Al-K) radiation (1486.6 eV), monochromatised by a double crystal monochromator and yielding a focused X-ray spot (elliptical in shape with a major axis length of 400 µm) were generated at 3 mA × 12 kV. The alpha hemispherical analyser operated in constant energy mode, scanning through the 200 eV energy to measure the entire energy band, and used 50 eV in a narrow scan to selectively measure specific elements. Avantage software was used to analyse the XPS data, and the smart background function was used to approximate the experimental background and calculate the elemental composition of the surface based on the peak area subtracted from the background. Charge compensation was achieved using the systems flood gun, which provides low-energy electrons and low-energy argon ions from a single source.

### 2.5. Scanning Electron Microscopy (SEM)

The surface modifications and morphological analysis were conducted with a Phenom ProX scanning electron microscope (Phenom World, Eindhoven, Netherlands). Samples were cut into square specimens of 2 mm x 2 mm. The microscope operates under high vacuum, using an electron beam at a potential of 5–15 keV, so that there is greater resolution of the image, going from micrometric to nanometric scale.

### 2.6. Sample Colour Measurements

The measurement of the colour difference of plasma treated leathers was carried out with the spectrophotometer CM-600d according to the standard EN ISO 22700:2020 [37]. This portable spectrophotometer is designed to assess the colour and appearance of samples of different sizes, including the surfaces of flat, shaped or curved objects. It has a fixed aperture of 8 mm and two measurement modes to suit the surface conditions of each sample: Specular Reflectance Included (SPINC) and Specular Reflectance Excluded (SPEX), the latter being used for measurement as it considers the surface finish of the sample. Measurements were made at three spots in the central part of the sample.

### 2.7. Surface Coating Thickness

The thickness of layer deposited onto the leather surface was determined according to the standard EN ISO 17186-method A [38]. A macm 1 rotary microtome (model 2030, Leica Reichert-Jung Biocut, Germany) was used with an optical microscope (model STD-18, Zeiss, Germany) equipped with a x 10 ocular with a graduated scale including a range of 2.6–261.9 µm and a x 16 magnification lens.

### 2.8. Water Contact Angles (WCA) Measurements

The hydrophobicity of the plasma coating was evaluated by determining the water contact angles. An optical contact angle measurement goniometer (Muver, Petrel, Spain) was used. This equipment has a thermostatised chamber that allows working in a saturated atmosphere, with an exhaustive control of the temperature at 25 °C. The equipment is provided with a vision system on a camera with a telecentric lens, which is backlit by a matrix of LEDs, and was used for droplet images captures. Deionised water was used as test liquid with a controlled volume of 4 µL by a syringe. Three drops were placed and measured at different points of the samples. Measurement procedure was performed as described in the standard EN 828-2013 [39]. The measurements were carried out at various times: 0 min, 5 min, 15 min, 25 min, 60 min and 90 min after plasma treatment to follow the wettability of the leather samples as a function of the time.

## 3. Results and Discussion

This section will provide a concise and precise description of the experimental results, their interpretation, as well as the experimental conclusions that can be drawn.

### 3.1. FTIR Analysis

**Figure 2:**
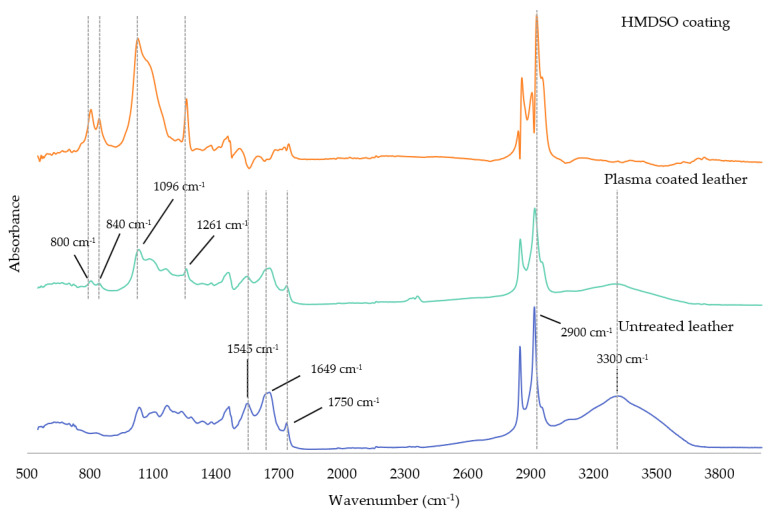
FTIR spectra of untreated leather sample, plasma coated leather and HMDSO plasma polymerised onto the leather surface.

FTIR was performed to further analyse the surface modifications of the plasma-coated leather samples with the non-polar 100% HMDSO, as well as to identify which functional groups contribute to the hydrophobic layer deposited. Figure 2 shows the FTIR spectra of the treated and untreated leather, as well as the spectrum corresponding to the HMDSO as precursor.

On the one hand, the FTIR spectrum of the natural leather as untreated sample showed sharp absorption peaks located at 1633 and 1649 cm^−1^ associated with the C=O amide in the peptide band (Amide I). The peak at 1545 cm^−1^ represented the N-H of Amide II and the peak at 1750 cm^−1^ corresponded to the C=O stretching due to the ester fatty acids. The absorption band between 2800 and 3000 cm^−1^ was related to -CH *stretching vibration mode (st)*. which was usually quite stable on the leather surface. In addition, amide A band appeared around 3300 cm^−1^ due to the stretching vibration of -NH groups and the conformation of the backbone, which was very sensitive to the strength of the hydrogen bonds [40].

On the other hand, the FTIR spectrum of the plasma-coated leather after activation, and subsequently, of the HMDSO polymerisation with oxygen and argon as carrier gases, respectively, showed the characteristic bands of the natural leather in addition to new bands corresponding to the deposited coating on the surface. Specifically, the bands between 2800–3000 cm^−1^ and 1261 cm^−1^ corresponded to C-H *st* and Si-(CH_3_) bending symmetric vibration (*δ_sy_*), respectively. The peak attributed to the Si-O-Si bonds appeared around 1096 cm^−1^, while the band at 840 and 800 cm^−1^ corresponded to the Si-C *st*and Si-(CH_3_) out-of-plane bending vibration (*γ*). The spectrum of the plasma-polymerised HMDSO layer deposited onto the leather surface was obtained by spectral subtraction, which confirmed the formation of a polysiloxane based layer on the leather surface [41,42,43]. Table 3 summarises the main characteristic bands observed in the mentioned samples [44,45,46]. In addition, the FTIR results were complemented with XPS analysis to comprehensively analyse the chemical groups created in the plasma-coated leather.

### 3.2. XPS Analysis

**Figure 3:**
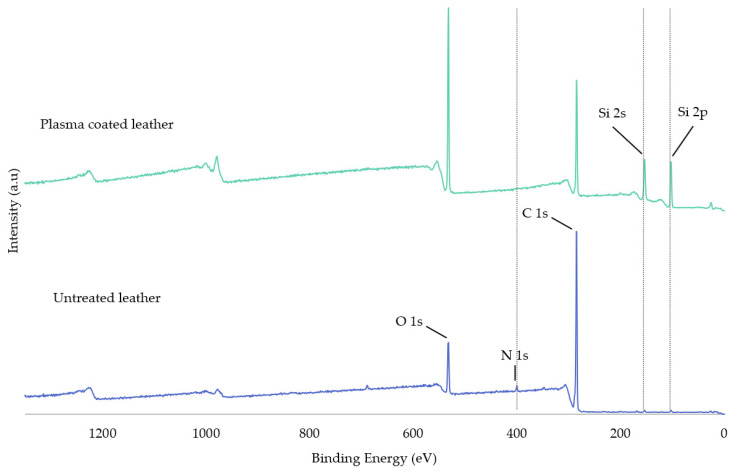
XPS-survey of untreated leather and plasma coated leather with HMDSO.

An XPS analysis was necessary to comprehensively analyse the chemical modifications on the outermost surface The surface chemistry of the untreated and plasma coated leather was analysed by X-ray photoelectron spectroscopy (XPS) to confirm the formation of silicon compounds, such as polysiloxanes, as a layer deposited on leather surface. Figure 3 shows the results of the XPS-survey of untreated leather and plasma coated leather. The leather surface was mainly composed of oxygen (O 1s), nitrogen (N 1s), carbon (C 1s) and low percentage of silicon (Si 2s and Si 2p), whose peaks were positioned at about 532, 400, 285, 154 and 103 eV. In the survey of plasma polymerised leather, the peaks appeared in the same positions for C, O, N and Si peaks, which highlights a considerable increase of oxygen and silicon peaks and a decrease of carbon and nitrogen, more noticeable in the latter, due to the plasma deposition of HMDSO [47,48].

For such purpose, the atomic percentages of the carbon, oxygen, nitrogen, and silicon components were determined and included in Table 4. It should be noted that in the deconvolution of the Si 2p spectrum, four contributions were shown in all samples corresponding to Si 2p3/2 and Si 2p1. Since they are doublet pair, to quantify only the most sensitive one, Si 2p3/2 was used. There was 1.12% silicon on the surface of the untreated sample, which may come from the manufacturing of leather or may be due to silicon existing in the analysis XPS chamber. Compared to the untreated sample, the atomic percentage of silicon in the HMDSO plasma treated sample increased up from 1.12 to 22.39% due to a high deposition of monomer which is composed of silicon components. Moreover, the atomic percentage of C decreased due to the removal and oxidation of organic compounds by the oxygen plasma used in the etching stage, and O increased after plasma treatment due to the introduction of new oxygenated by etching and siliconised molecules deposited on the leather surface, and because of their presence in the polysiloxane composition. The nitrogen (1.33%) that appeared in the untreated leather is due to the proteins that constitute the original leather. However, in the HMDSO treated sample, 0.25% of nitrogen was observed. The detection of this small amount of several nitrogenous functional groups in the plasma coated leather could be due to contamination because of the 3–7 nm depth the XPS reaches and the thickness of the deposited HMDSO layer being greatly higher, as can be seen in Section 3.5. Therefore, the amount of nitrogen obtained in the coated sample can be considered negligible, indicating that no signal from the XPS-based substrate has been obtained and uniform coverage has been made on the substrate during plasma deposition. Furthermore, the atomic ratios showed an increase in the percentage of oxygen and silicon and a decrease in the atomic percentage of carbon due to the named chemical modifications [14,15,49].

**Figure 4:**
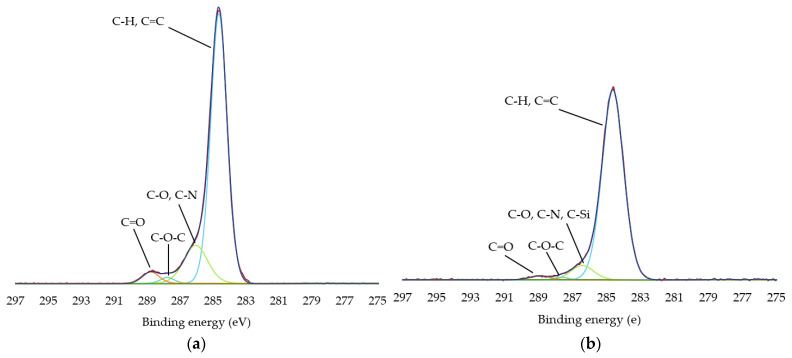

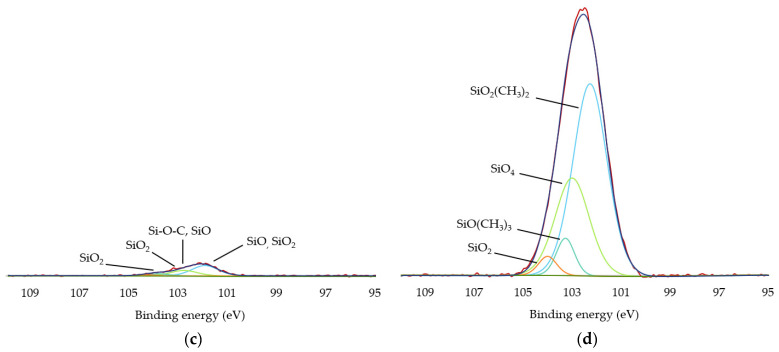
High-resolution peaks of HMDSO film deposited on leather. (**a**) C 1s of untreated sample; (**b**) C 1s of plasma coated leather; (**c**) Si 2p of untreated sample; (**d**) Si 2p of plasma coated leather.

Figure 4 shows the deconvoluted C1s and Si 2p peak spectra of the leather with and without plasma deposition, and Table 5 includes the functional group contents of carbon and silicon. The decomposed bands were assigned to the appropriate functional groups. The resolution of the C 1s peak curve of the untreated leather was fitted with four peaks: one large peak was located at about 284.64 eV, due to the C-H or C=C bond; the other peak was found at about 286.05 eV corresponding to the C-O or C-N bond; and two small peaks were about 287.77 eV, due to the C-O=O, and about 288.78 eV the C=O bond appeared. In the plasma coated samples, the C 1s peak was also the same as in the untreated leather, but the intensity was lower due to the HMDSO coating, and a new contribution appeared at 286.42 eV corresponding to the C-Si bond which was due to the methyl groups present in the decomposed HMDSO. It was also possible to perform curve resolution of the Si 2p peaks to obtain more information about the chemical bonds in the samples. The deconvolution of Si 2p spectra of the untreated leather showed low content of silicon oxides (SiO) at 101.90 and 102.76 eV, silicon oxycarbide (Si-O-C) at 102.76 eV and silicon dioxide (SiO_2_) at 101.90, 103.75 and 104.28 eV. The silicon oxides could have formed on the surface of the leather due to its manufacture and the XPS chamber. The Si 2p peaks of the plasma coated leather showed SiO_2_(CH_3_)_2_, SiO_4_, SiO(CH_3_)_3_ and SiO_2_ units at approximately 102.27, 103.00, 103.27 and 104.00 eV, respectively. This result was due to the formation of a new layer of silicon compound on the surface by HMDSO plasma deposition. The high portion of SiO_2_(CH_3_)_3_ indicates that the film had a structure like polydimethylsiloxane (PDMS). The presence of SiO(CH_3_)_3_ is the oxidation product of PDMS. The inorganic structures SiO_2_ and SiO_4_ represent complete oxidation at the deconvolution peak. These results support the formation of a SiO_x_C_y_H_z_ film with an organic structure using Ar as a carrier gas, as well as an inorganic structure through the O_2_ etching stage [15,17,49,50,51].

### 3.3. SEM Analysis

**Figure 5:**
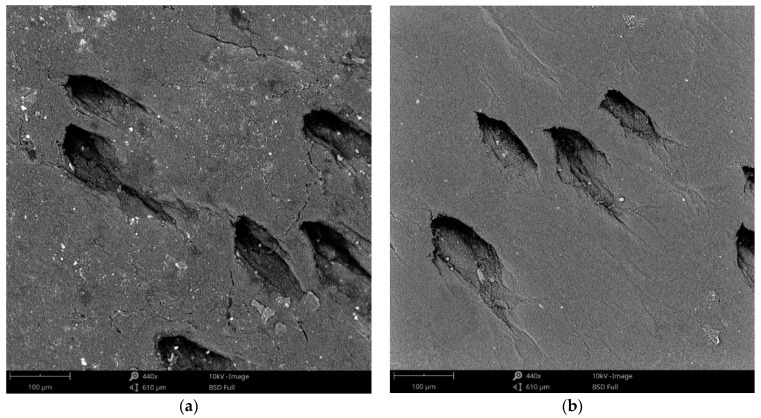
SEM images obtained before and after plasma polimerisation (440×). (**a**) Untreated leather; (**b**) Plasma coated leather.

The morphology of the leather surface before and after HMDSO plasma deposition was analysed by means of scanning electron microscopy according to the images shown in Figure 5. The untreated sample appears with small cracks and some white spots; such surface irregularities are typical in materials of natural origin such as leather, and the dark, rough pores of the leather are also visible. In the plasma deposited material, the gaps or pinholes caused by the animal hair follicles were partially covered by the plasma coating making them smaller. It was observed that a smooth, clean, thin film was formed on the remaining parts of the substrate, which covered the cracks. These modifications to the surface of the HMDSO plasma deposition treated material resulted in the coating being uniformly deposited creating an ultra-thin hydrophobic layer on the leather [52].

### 3.4. Colour Change Measurements

**Figure 6:**
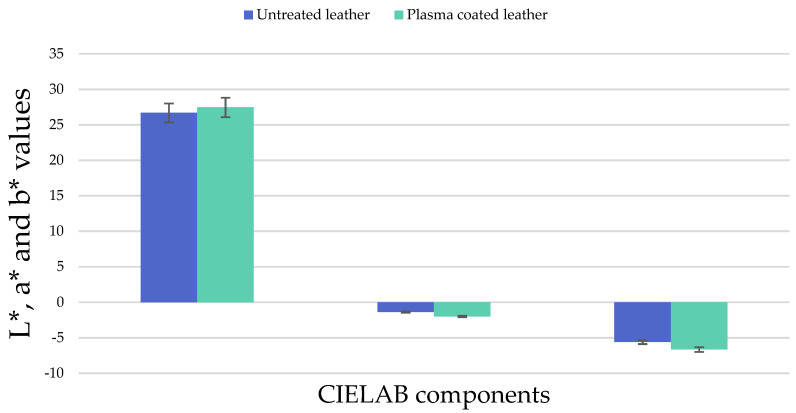
L*, a* and b* values for both untreated and plasma treated leather.

**Figure 7:**
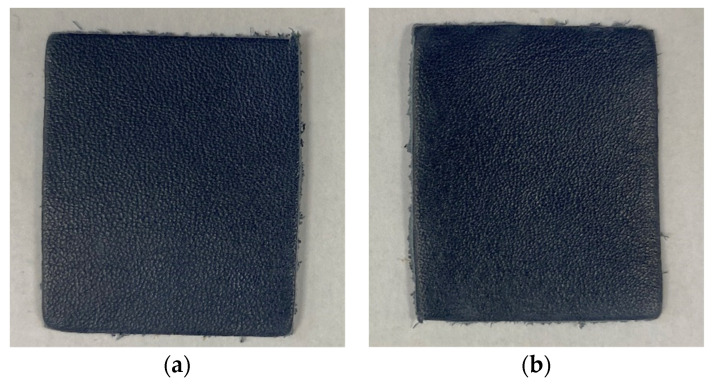
Pictures of (**a**) untreated and (**b**) plasma coated leather samples.

The influence of the plasma treatment on the colour was measured in CIELAB L*, b* and a* values, as shown in Figure 6. The CIELAB colour system quantifies the relationship of the colours on three axes: L* indicates the lightness, and a* and b* are chromatic coordinates corresponding to the colours red/green and yellow/blue, respectively. In the component values obtained, no significant changes were observed depending on the plasma treatment and the untreated sample. It can be highlighted that the HMDSO coating deposited by plasma polymerisation on the leather sample does not affect neither the colour nor the surface appearance of the original pigment of the leather, as can be seen in Figure 7, according to the requirement that there must be a difference between the values ≤ 2.5 established by INESCOP’s upper materials laboratory based on its experience [53].

### 3.5. Surface Coating Thickness

**Figure 8:**
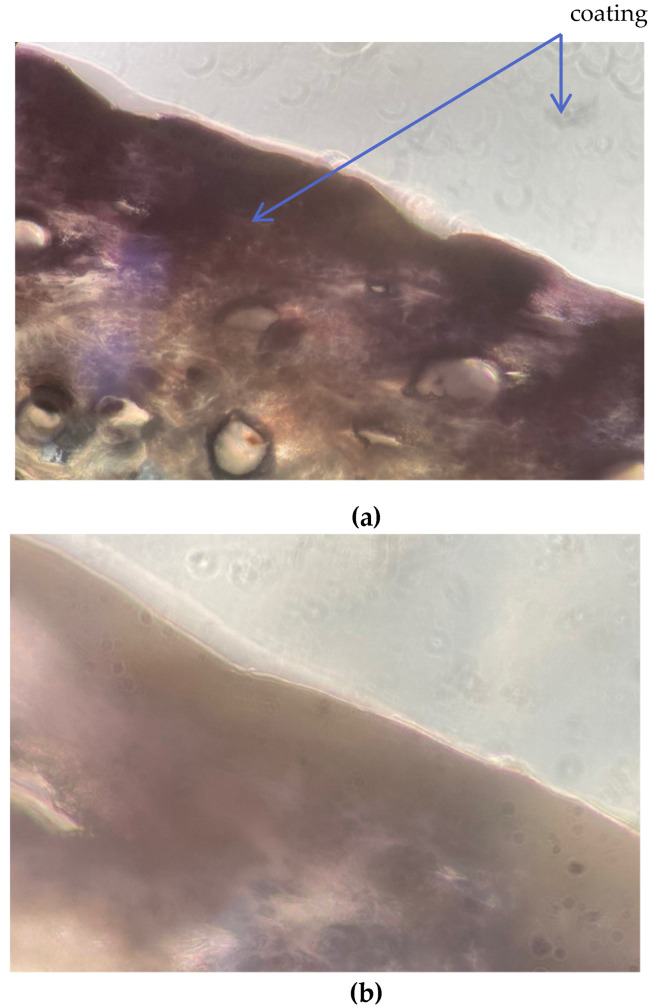
Cross-section images of plasma coated leather of HMDSO at 16× (**a**) and magnified at 40× (**b**).

The thickness of the HDMSO film deposited by plasma on the leather samples was measured by a manual rotary microtome coupled to an optical microscope according to the standard EN ISO 17186-method A. Table 6 includes the different thickness values determined, which have been obtained from three cuts of leather and, in each cut, 3 measurements have been made. The result is the average of the nine measurements. The thickness of the untreated leather was less than that of the plasma coated leather films. The HMDSO coating formed was 600 nm (see Figure 8), which is considered very small compared to a coating thickness on the aniline finish leather [15]. In addition, it can still be considered leather as the coating limit is 150,000 nm according to European regulations and standards [54,55,56].

### 3.6. Water Contact Angle (WCA) Measurements

**Figure 9:**
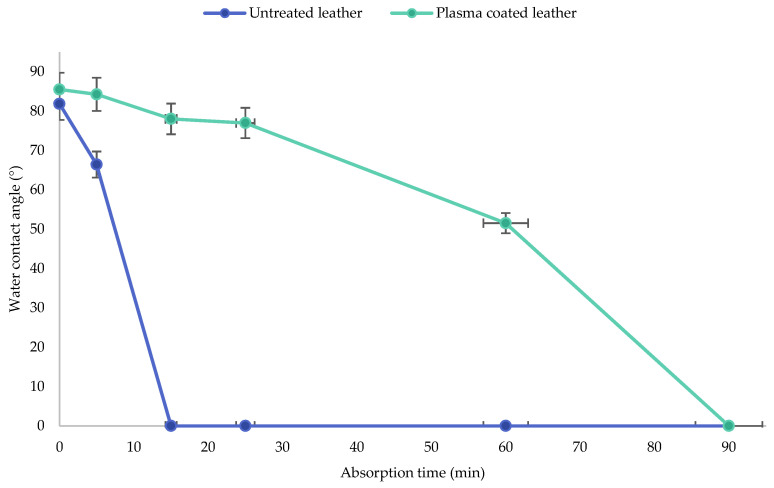
Evolution of wettability of untreated and plasma treated samples over time.

The results of contact angle measurements with distilled water and absorption times of the plasma-treated and untreated leather are shown in Table 7. In addition, the evolution of wettability of untreated and plasma-treated samples over time has been represented in Figure 9.

For natural leather as untreated sample, the water contact angle decreased from 81.83° to 0.33° within the first 15 min, time in which the water droplets had already been completely absorbed and entirely spread on the sample due to the hydrophilic nature of the natural leather. However, in the treated sample, the evolution of the contact angle as a function of time was quite different. Within the first 15 min, the water contact angle slightly decreased from 85.48° to 78.25°. After 25 min, the water droplets remained again on the surface with a similar contact angle of 76.97°. After 90 min, the contact angle reached 0.00° because the evaporation and contraction of the droplets occurred. Therefore, it can be said that the HMDSO plasma polymerised coating on leather provided high hydrophobic performance to leather, which exceeded the absorption time of 60 min [57].

The results of the wettability study showed that the surface hydrophobicity of the leather samples is significantly improved. This result can be attributed to the hydrophobic surface formed by plasma deposition of the silicon compound of polysiloxane. Plasma polymerisation of HMDSO with the application of 150 W plasma power with an initial surface activation process of 300 s with oxygen and alternating treatments of 12 s oxygen etching and 300 s argon coating resulted in a noticeable hydrophobic film coating on the leather surface [58,59]

## 4. Conclusions

In this study, the surface modifications of natural leather samples, a material commonly used for footwear applications, by plasma polymerisation with a 100% HMDSO composition have been evaluated by means of different experimental techniques. According to the results, the combination of optimised plasma activation and etching pre-treatment, both with O_2_ and coating treatment with HMDSO/Ar, improved the hydrophobicity of the surface due to the introduction and deposition of silane groups on the leather surface. More specifically, the formation of an ultra-thin hydrophobic layer of polysiloxane nature and completely deposited by low-pressure plasma led to high water contact angles and absorption times compared to the natural and untreated leather. Most importantly, the HMDSO plasma coating deposited on the leather samples does not affect the original pigment of the leather, neither the colour nor the surface appearance and feel.

It can be concluded that plasma deposition of HMDSO at low pressure showed promising results to provide natural leather with water repellence for footwear applications. Plasma hydrophobic coatings may be used as a more sustainable alternative to replace conventional treatments currently used based on halocarbons and organic solvents.

Finally, it should be noted that this study contributes greatly to the three pillars of the Sustainable Development Goals (SDGs): the economic, social and environmental objectives set by the European Green Deal and its Circular Economy Plan, enabling thus the footwear sector to move increasingly towards a production model based on sustainability and automation, and contributing to the flexibility of processes and the modernisation of the industry by increasing its resilience [60].

## Figures and Tables

**Table 1:** Physical and mechanical properties of leather.

Material	Tensile Strength (MPa)	Elongation (%)	Dichloromethane Extractable Matter (wt %)	Ash Content at 950 °C (wt %)
Leather	16.2	57	5.85	4.3

**Table 2:** Plasma operating conditions of the plasma deposition process.

Plasma Stages	Gas	Time (s)	Power (W)	Pressure (Pa)	Monomer	Dossing Pump (µL/s)
Activation	O_2_	300	150	300	-	-
Polymerisation	Ar	300	150	300	HMDSO	0.5
Etching	O_2_	12	150	300	-	-
Polymerisation	Ar	300	150	300	HMDSO	0.5
Etching	O_2_	12	150	300	-	-
Polymerisation	Ar	300	150	300	HMDSO	0.5

**Table 3:** Assignments of the main FTIR absorption bands observed for the spectra in Figure 2.

Wavenumber	Assignment
800 cm^−1^	(Si-)CH_3_ *γ*
840 cm^−1^	Si-C *γ*
1096 cm^−1^	Si-O-Si
1261 cm^−1^	(Si-)CH_3_ *δ_sy_*
1545 cm^−1^	N-H (Amide II)
1633–1649 cm^−1^	C=O (Amide I)
1750 cm^−1^	C=O ester
2800–3000 cm^−1^	-CH *st*
3300 cm^−1^	-NH (Amide A)

**Table 4:** Elemental compositions of untreated and HMDSO plasma deposited leather sample.

Sample	Atomic Percentages (%)				Atomic ratio	
	C	O	N	Si	Si/C	Si/O
Untreated leather	84.87	12.44	1.33	1.12	0.01	0.09
Plasma coated leather	48.81	27.70	0.25	22.39	0.45	0.80

**Table 5:** Atomic percentages (at. %) of chemical species at C 1s and Si 2p peaks of the untreated and plasma coated leather determined by XPS.

	Untreated Leather				Plasma Coated Leather		
Element	Chemical State	Binding Energy (eV)	At. (%)	Element	Chemical State	Binding Energy (eV)	At. (%)
C 1s	C-H, C=C	284.64	67.05	C 1s	C-H, C=C	284.62	44.77
	C-O, C-N	286.05	13.63		C-O, C-N, C-Si	286.42	3.67
	C-O-C	287.77	1.21		C-O-C	287.66	0.35
	C=O	288.78	2.98		C=O	288.94	0.86
Si 2p	SiO or SiO_2_	101.90	0.98	Si 2p	SiO_2_(CH_3_)_2_	102.27	20.29
	Si-O-C, SiO	102.76	-		SiO_4_	103.00	-
	SiO_2_	103.75	0.14		SiO (CH_3_)_3_	103.27	2.10
	SiO_2_	104.28	-		SiO_2_	104.00	-

**Table 6:** Thickness values of untreated, plasma treated samples and plasma polymerised HMDSO coating.

Sample	Thickness (nm)
Untreated leather	3700
Plasma coated leather	4300
HMDSO coating	600

**Table 7:** Absorption time and contact angle in plasma treated and untreated leather samples.

Absorption Time (min)	Water Contact Angle (WCA)	
	Untreated Leather	Plasma Coated Leather
t = 0 min	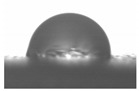	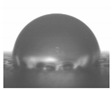
	81.83° ± 1.58	85.48 ± 0.76
t = 5 min	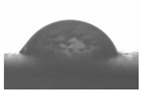	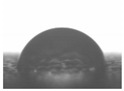
	66.41° ± 2.66	83.43° ± 0.77
t = 15 min	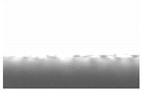	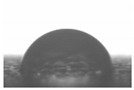
	0.33° ± 0.29	78.25° ± 0.72
t = 25 min	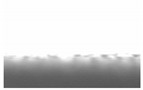	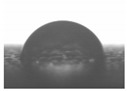
	0.00° ± 0.00	76.97° ± 2.38
t = 60 min	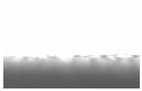	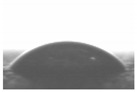
	0.00° ± 0.00	51.52° ± 0.46
t = 90 min	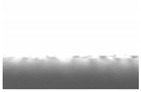	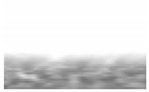
	0.00° ± 0.00	0.00° ± 0.00

## Data Availability

The data presented in this study are available upon request from the corresponding author.
